# Association between human immunodeficiency virus diagnosis and risk of penoscrotal conditions in adult males: A propensity‐matched retrospective cohort study

**DOI:** 10.1002/bco2.70291

**Published:** 2026-10-14

**Authors:** Hossein A. Zolfaghari, Melissa Nanotkar, Juan Riestra, Aleksandar Popovic, Diba Atar, Amjad Alwaal

**Affiliations:** ^1^ Department of Surgery Urology Division Rutgers‐New Jersey Medical School Newark New Jersey USA; ^2^ Department of Medical Education Penn State College of Medicine Hershey Pennsylvania USA

**Keywords:** cohort studies, epidemiological studies, epididymo‐orchitis, HIV, phimosis, priapism

## Abstract

**Objectives:**

This study aimed to examine the temporal association between human immunodeficiency virus (HIV) diagnosis and subsequent development of penoscrotal conditions including priapism, phimosis, epididymo‐orchitis, testicular torsion and paraphimosis.

**Patients and methods:**

A propensity‐matched retrospective cohort study was conducted using the TriNetX database. Adult males with HIV (B20) were matched 1:1 to controls on age and race. Outcomes were assessed over a 10‐year follow‐up period using the Cox proportional hazards model.

**Results:**

Following matching, cohort sizes ranged from 48 615 to 65 423 patients per group. HIV diagnosis was associated with significantly increased rates of priapism (HR 1.892, 95% CI 1.115–3.210, *p* = 0.0162), phimosis (HR 2.364, 95% CI 1.631–3.428, *p* < 0.0001) and epididymo‐orchitis (HR 2.346, 95% CI 2.116–2.602, *p* < 0.0001). No significant association was observed for testicular torsion (HR 1.579, 95% CI 0.756–3.295, *p* = 0.2200). Paraphimosis could not be evaluated in the corrected analysis due to insufficient data.

**Conclusion:**

HIV diagnosis was associated with significantly elevated rates of priapism, phimosis and epididymo‐orchitis, while no significant association was observed for testicular torsion. These findings are hypothesis‐generating, and prospective studies with more granular clinical data are needed to confirm and characterize these associations.

## 1 INTRODUCTION

Human immunodeficiency virus (HIV) is a chronic disease that leads to widespread immunosuppression, which results in a variety of clinical manifestations one of which is sexual dysfunction.^1^, ^2^ Approximately half of individuals with HIV experience issues such as loss of libido.^2^, ^3^ Prevalence of erectile dysfunction is also significantly increased in HIV populations compared to HIV‐negative populations.^2^ Proposed mechanisms underlying sexual dysfunction in HIV‐positive populations include endocrine dysfunction, as increased hypogonadism in patients with HIV could lead to lower testosterone levels through HIV‐associated visceral fat accumulation.^4^ Usage of protease inhibitors as part of antiretroviral therapy (ART) is also associated with decreased erectile function as well as decrease in sexual interest, but this conclusion remains disputed with contradictory results in certain studies.^2^, ^5^, ^6^, ^7^, ^8^ Furthermore, the highly increased prevalence of anxiety and depression in HIV patients has been correlated with increased rates of erectile dysfunction.^2^, ^9^

While development of chronic sexual dysfunction in the context of HIV has been extensively documented, the association between HIV and penoscrotal conditions remains understudied. These conditions, including priapism, phimosis, epididymo‐orchitis, testicular torsion and paraphimosis, represent a range of genitourinary pathologies with distinct mechanical, vascular and infectious etiologies and may result in significant morbidity including erectile dysfunction, infertility and tissue loss if unrecognized or inadequately treated.^10^, ^11^, ^12^, ^13^, ^14^ This study originated from an interest in the association of acute penoscrotal emergencies with HIV, namely, priapism, testicular torsion and paraphimosis, and was subsequently broadened to include phimosis and epididymo‐orchitis, two additional penoscrotal conditions that can each present acutely or require urgent evaluation. Examining this range of conditions, rather than a single pathology, allows assessment of whether HIV's association with genitourinary disease reflects a broad‐based effect or is restricted to specific pathophysiological pathways.

Understanding the temporal association between HIV and these penoscrotal conditions is clinically relevant given the widespread immunosuppression and genitourinary vulnerability characteristic of HIV infection. This study aims to examine the association between HIV diagnosis and subsequent development of priapism, phimosis, epididymo‐orchitis, testicular torsion and paraphimosis using a propensity‐matched retrospective cohort study with the TriNetX database. These findings are intended to be hypothesis‐generating, with the goal of informing future prospective investigation into HIV‐related genitourinary morbidity.

## 2 PATIENTS AND METHODS

A retrospective cohort study was conducted using the TriNetX database, a global health research database with de‐identified electronic medical record data from healthcare organizations worldwide. Adult males from both academic and nonacademic United States health centres who were diagnosed with HIV (ICD‐10: B20) were included. The index event was defined as the date of HIV diagnosis for the HIV cohort and the date of a qualifying ambulatory visit for the control cohort. Outcomes were assessed over a 10‐year observation window beginning 1 day after the index event. Patients who were lost to follow‐up, died or reached the end of available data prior to 10 years were censored at their last recorded clinical fact, as handled automatically by the TriNetX Cox proportional hazards model. Controls were propensity matched 1:1 on age and race using the TriNetX platform algorithm, which employs 1:1 greedy nearest‐neighbour propensity score matching. Propensity scores were estimated using logistic regression. Cohort balance was assessed using standardized mean differences (SMDs), with SMD < 0.1 considered adequate balance.

Exclusion criteria for the risk of developing priapism included sickle cell disease (ICD‐10: D57.0, D57.1, D57.2, D57.4, D57.8), leukaemias (C95), thalassemias (D56), Peyronie's disease (N48.6) and male erectile dysfunction (N52). Primary outcome was defined as at least one priapism (N48.30) event within 10 years following the index event.

Exclusion criteria for the risk of developing phimosis included balanitis (ICD‐10: N48.1), balanoposthitis (N47.6), lichen planus (L43), psoriasis (L40), atopic dermatitis (L20), type 2 diabetes mellitus (E11), lichen sclerosus (L90.0), pemphigus (L10) and Stevens–Johnson Syndrome (L51.1). Primary outcome was defined as at least one phimosis event (N47.1) within 10 years following the index event.

Exclusion criteria for the risk of developing epididymo‐orchitis included mumps (B26.0) brucellosis (A23), as independent infectious etiologies. Primary outcome was defined as at least one epididymitis (N45.1), orchitis (N45.2) or epididymo‐orchitis (N45.3) event within 10 years following the index event.

Exclusion criteria for the risk of developing testicular torsion included prior testicular torsion events, undescended and/or ectopic testicles (ICD‐10: Q53), orchiectomy (54 535, 54 522, 54 530, 1 008 565, 54 520), congenital abnormalities of the testes and scrotum including bell‐clapper deformity (Q55.29) and any malignant neoplasm of the testis (C62). Primary outcome was defined as at least one testicular torsion event (N44.00) within 10 years following the index event.

Exclusion criteria for the risk of developing paraphimosis included hypospadias (ICD‐10: Q54), epispadias (Q64.0), phimosis (N47.1), balanitis (N48.1), balanoposthitis (N47.6) and kidney failure (N19). Primary outcome was defined as at least one paraphimosis event (N47.2) within 10 years following the index event.

All analyses were performed using the TriNetX platform, which uses the Cox proportional hazards model to assess risk over a 10 year follow‐up period. The number of patients in each cohort with each outcome, survival probability, hazard ratio and 95% C.I. were determined for each analysis. Statistical significance was defined as *p* < 0.05. All data utilized were de‐identified without data export and therefore did not require institutional review board approval.

## 3 RESULTS

Baseline characteristics of the propensity‐matched cohorts are presented in Table 1. Across all analyses, matched cohort sizes ranged from 48 615 to 65 423 patients per group, with follow‐up extending to 10 years from the index event. Following matching, all cohorts demonstrated excellent balance on age and race, with SMDs below 0.1 for all variables.

**TABLE 1:** Baseline characteristics of propensity‐matched cohorts.

Outcome	*N* per cohort^a^	Mean current age ± SD (years)	Mean age at index ± SD (years)	White (%)	Black or African American (%)
Priapism	51 803	56.1 ± 13.1	42.3 ± 12.9	45.6	36.9
Phimosis	48 615	55.3 ± 12.9	41.5 ± 12.7	45.9	37.3
Epididymo‐orchitis	65 423	56.6 ± 12.9	42.9 ± 12.8	46.1	38.4
Testicular torsion	61 894	56.8 ± 12.9	43.0 ± 12.7	46.6	37.3
Paraphimosis	60 862	56.6 ± 12.9	42.8 ± 12.7	46.4	37.8

*N* per cohort refers to matched patients in each group.

Hazard ratios for each penoscrotal condition over the 10‐year follow‐up period are summarized in Table 2. HIV diagnosis was associated with significantly increased rates of priapism (HR 1.892, 95% CI 1.115–3.210, *p* = 0.0162; 44 vs 20 events), phimosis (HR 2.364, 95% CI 1.631–3.428, *p* < 0.0001; 104 vs 38 events) and epididymo‐orchitis (HR 2.346, 95% CI 2.116–2.602, *p* < 0.0001; 1321 vs. 496 events). HIV diagnosis was not associated with a statistically significant increase in risk for testicular torsion (HR 1.579, 95% CI 0.756–3.295, *p* = 0.2200; 20 vs. 11 events). Paraphimosis could not be evaluated in the corrected analysis as event counts fell below the TriNetX minimum reporting threshold.

**TABLE 2:** Hazard ratios for penoscrotal conditions over 10‐year follow‐up^a^.

Outcome	Hazard ratio	95% CI	Log‐rank *p*‐value	HIV events (*n*)	Control events (*n*)
Priapism	**1.892**	1.115–3.210	0.0162	44	20
Phimosis	**2.364**	1.631–3.428	<0.0001	104	38
Epididymo‐orchitis	**2.346**	2.116–2.602	<0.0001	1321	496
Testicular torsion	1.579	0.756–3.295	0.2200	20	11

Paraphimosis results are not reported. Outcome event counts fell below the TriNetX minimum reporting threshold of 10 patients in both cohorts, precluding statistical comparison. Bold indicates statistical significance (*p* < 0.05).

A subgroup analysis stratifying HIV patients by antiretroviral treatment status was attempted for all outcomes; however, event counts in both subgroups consistently fell at or below the TriNetX minimum reporting threshold, precluding reliable statistical comparison.

## 4 DISCUSSION

In this propensity‐matched retrospective cohort study, HIV diagnosis was associated with significantly increased rates of priapism, phimosis and epididymo‐orchitis over a 10‐year follow‐up period, while no significant association was observed for testicular torsion. Paraphimosis could not be evaluated in the corrected analysis. To our knowledge, this is the first large‐scale propensity‐matched retrospective cohort study to systematically examine the temporal association between HIV diagnosis and this range of penoscrotal conditions.

HIV diagnosis was associated with an increased rate of priapism (HR 1.892, 95% CI 1.115–3.210). This association is biologically plausible given HIV's role in endothelial dysfunction and hypercoagulability, both of which may impair venous outflow within the corpora cavernosa and reduce nitric oxide bioavailability, creating conditions favourable for sustained pathological erection.^15^, ^16^ Prior literature has also implicated ART as a potential independent contributor to priapism risk, though a subgroup analysis by treatment status was not feasible in this dataset due to event counts falling below the TriNetX reporting threshold.^10^, ^17^

HIV diagnosis was associated with an increased rate of phimosis (HR 2.364, 95% CI 1.631–3.428). This association is biologically plausible through two mechanisms: HIV‐induced immunosuppression may predispose the penile epithelium to lichen sclerosus, the dominant pathological driver of adult acquired phimosis, found in 14%–95% of foreskins removed for phimosis^18^, and HIV‐related immunosuppression increases susceptibility to recurrent balanitis and balanoposthitis, which drive progressive preputial scarring through cycles of microbial infection and local inflammation.^11^, ^19^, ^20^

HIV diagnosis was associated with an increased rate of epididymo‐orchitis (HR 2.346, 95% CI 2.116–2.602). This association is biologically plausible through two complementary mechanisms. First, HIV‐related immunosuppression directly impairs local genitourinary defences, increasing susceptibility to the bacterial and sexually transmitted pathogens most commonly responsible for epididymo‐orchitis.^12^ Second, HIV‐positive populations experience significantly elevated rates of *Chlamydia trachomatis* and *Neisseria gonorrhoeae*, which account for the majority of epididymo‐orchitis cases in men under 40, and this elevated STI burden is itself a consequence of HIV‐related immune dysregulation and the associated behavioural risk profile.^21^, ^22^ It is important to note that sexually transmitted infections were not excluded as a covariate in this analysis, and the relative contributions of direct immunosuppression versus STI‐mediated pathways to the observed association remain an important area for future investigation.

These findings suggest that clinicians caring for HIV‐positive men may benefit from awareness of the elevated rates of priapism, phimosis and epididymo‐orchitis observed in this population. The associations identified across three distinct penoscrotal conditions, each with different underlying mechanisms, highlight the broad impact that HIV‐related immunosuppression may have on genitourinary health. However, given the observational nature of this study, these findings should be interpreted as hypothesis‐generating rather than as a basis for definitive clinical recommendations.

In contrast to priapism, phimosis and epididymo‐orchitis, HIV was not associated with a statistically significant increase in risk for testicular torsion (HR 1.579, 95% CI 0.756–3.295, *p* = 0.2200). Testicular torsion is primarily a mechanical and anatomical event driven by predisposing structural factors such as bell‐clapper deformity and is therefore unlikely to be substantially modified by the systemic inflammatory and vascular pathology characteristic of HIV.^23^ Notably, the absence of a significant association for testicular torsion, a mechanically driven condition, while significant associations were observed for immunosuppression‐mediated conditions, suggests that these findings reflect specific biological pathways rather than a general detection bias. Paraphimosis could not be evaluated in the corrected analysis as event counts fell below the TriNetX reporting threshold.

## 5 LIMITATIONS

This study has several important limitations. As a retrospective observational cohort study, these findings demonstrate temporal associations but cannot establish causality. Additionally, despite propensity score matching on age and race, residual confounding from unmeasured variables, including HIV disease severity, CD4 count at diagnosis, viral load, circumcision status, sexual behaviour and socioeconomic factors, may influence the observed associations. Circumcision status is a particularly relevant unmeasured confounder for phimosis, as uncircumcised men are at substantially higher baseline risk and circumcision rates differ between HIV‐positive and HIV‐negative populations; however, circumcision status is not available within the TriNetX database and could not be accounted for in this analysis. As with any retrospective database study, it was not feasible to exclude every possible confounder associated with each outcome; for example, medication and substance exposures known to independently cause priapism, such as intracavernosal injection therapy and vasoactive substances, including cocaine, were not applied as exclusion criteria in this analysis. Future studies should continue to parse out the relative contributions of these established risk factors alongside HIV status.

Additionally, the index event differed between cohorts, with the HIV cohort indexed at date of HIV diagnosis and the control cohort at a generic ambulatory visit. HIV‐positive patients typically enter a system of more frequent structured follow‐up after diagnosis, which may increase the likelihood of detecting and coding subsequent conditions, representing a form of detection bias that cannot be fully corrected within the TriNetX platform. Finally, absolute event counts were small for priapism (44 vs. 20 events) and testicular torsion (20 vs. 11 events), limiting statistical power for these outcomes, and a subgroup analysis stratifying by antiretroviral treatment status was not feasible as event counts in both subgroups consistently fell below the TriNetX minimum reporting threshold.

## 6 FUTURE DIRECTIONS

This study opens several avenues for future investigation. Prospective studies with clinical data, including CD4 count, viral load, ART regimen, circumcision status and STI history, are needed to better characterize the associations observed and determine whether earlier ART initiation or viral suppression modifies risk for these penoscrotal conditions. Given the mechanistic role hypothesized for ART medications in priapism risk, future studies should examine individual ART drug classes separately to identify whether specific regimens confer greater or lesser risk. For epididymo‐orchitis specifically, future studies should stratify by STI status to better characterize the relative contributions of direct immunosuppression versus STI‐mediated pathways to the elevated risk observed. The relationship between HIV and lichen sclerosus warrants dedicated investigation, as this pathway may represent a significant and underrecognized source of morbidity in HIV‐positive men. Larger multicenter datasets with sufficient event numbers would also enable powered subgroup analyses by treatment status, race and age, which were not feasible in the present study.

## 7 CONCLUSIONS

In this propensity‐matched retrospective cohort study, HIV diagnosis was associated with significantly increased rates of priapism (HR 1.892, 95% CI 1.115–3.210), phimosis (HR 2.364, 95% CI 1.631–3.428, and epididymo‐orchitis (HR 2.346, 95% CI 2.116–2.602) over a 10‐year follow‐up period, while no significant association was observed for testicular torsion. Paraphimosis could not be evaluated in the corrected analysis. The specificity of associations for immunosuppression‐mediated conditions, while testicular torsion, a mechanically driven condition, showed no significant association, suggests that these findings reflect biologically plausible pathways rather than a general detection bias. These findings are exploratory and hypothesis‐generating, and prospective studies with more granular clinical data, including CD4 count, viral load, ART regimen, circumcision status and STI history, are needed to confirm and characterize these associations.

## AUTHOR CONTRIBUTIONS

**Hossein A. Zolfaghari**: Conceptualization; methodology; data curation; formal analysis; writing—original draft; writing—review and editing. **Melissa Nanotkar**: Conceptualization; data curation; formal analysis; writing—original draft; writing—review and editing. **Juan Riestra**: Writing—review and editing. **Aleksandar Popovic**: Writing—review and editing. **Diba Atar**: Writing—review and editing. **Amjad Alwaal**: Writing—review and editing; supervision.

## CONFLICT OF INTEREST STATEMENT

All authors certify that they have no affiliations with or involvement in any organization or entity with any financial interest or nonfinancial interest in the subject matter or materials discussed in this manuscript.

## Data Availability

The data that support the findings of this study are available from the authors but restrictions apply to the availability of these data, which are from the TriNetX US Collaborative Network and so are not publicly available. Data are, however, available from the authors upon reasonable request.
